# A rare case of nitrofurantoin‐induced acute lung injury

**DOI:** 10.1002/ccr3.3314

**Published:** 2020-09-16

**Authors:** Rehan Karmali, Kyle Stawitzky, Isrin Srisethnil, Kaitlyn Simpson

**Affiliations:** ^1^ Touro College of Osteopathic Medicine Middletown New York USA; ^2^ Department of Family Medicine Garnet Health Medical Center Middletown New York USA

**Keywords:** acute lung injury, drug‐induced pulmonary toxicity, nitrofurantoin

## Abstract

Nitrofurantoin is a common treatment for urinary tract infections. Acute lung injury resulting from nitrofurantoin is a rare, life‐threatening complication with women being at greater risk. Symptoms include respiratory distress with fevers, rash, eosinophilia, and new‐onset atrial fibrillation. Treatment includes discontinuing the drug and possibly glucocorticoids for persistent oxygen demand.

## Graphical abstract

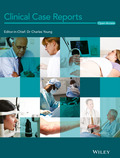

## 1 INTRODUCTION

Since its approval by the FDA in 1953, nitrofurantoin has been commonly prescribed as a treatment for lower urinary tract infections.^1^ Besides allergies, one known complication of nitrofurantoin use is pulmonary toxicity in both the acute and chronic settings.^2^ Acute pulmonary toxicity is categorized as a hypersensitivity reaction with common symptoms being fever, dyspnea, and cough with eosinophilia seen in lab findings. The chronic form is better characterized by direct tissue damage causing dyspnea, a dry cough, and fatigue.^3^ The most common pulmonary complication is the acute hypersensitivity reaction with an incidence rate of approximately 1 in 5000 patients after first‐time nitrofurantoin use.^1^ Here we present the case of a 79‐year‐old female who was diagnosed with acute nitrofurantoin‐induced lung injury after an extensive workup ruling out cardiac, pulmonary, and cancerous etiologies.

## 2 CASE PRESENTATION

The patient is a 79‐year‐old‐female with a past medical history of coronary artery disease (CAD) status/post 6 stents (last in 2006), hypertension (HTN), hyperlipidemia (HLD), and type 2 diabetes (T2DM) who presented to the emergency department (ED) with mid‐sternal nonradiating chest pain that became generalized across her chest wall. The pain started 6 hours before presentation when she was on a 3‐hour drive home after visiting her daughter. She initially went to Urgent Care where she took some aspirin, but her pain did not improve prompting her visit to the ED. She reported some associated nausea, but otherwise denied fevers, chills, shortness of breath, abdominal pain, vomiting, diarrhea, headache, tingling, or dizziness. Her chronic medications included amlodipine, aspirin, atorvastatin, losartan‐hydrochlorothiazide, metformin, and metoprolol. She was started on nitrofurantoin, twice daily, 2 days prior to presentation for a urinary tract infection (UTI), which was discontinued on admission. She did not use any supplemental oxygen at home and her most recent cardiac stress test 1 year prior to admission was normal. On physical exam, she had a regular heart rate and rhythm without any murmurs, rubs, or gallops. Her lungs were clear to auscultation bilaterally without any wheezing, rales, or rhonchi. She did not have any extremity edema, cyanosis, calf tenderness, and had 2+ pulses bilaterally. Her abdomen was soft and nondistended and she was alert and oriented without any focal neurological deficits. Her oxygen saturation was 89% on room air and remaining vital signs were within normal limits.

**FIGURE 1:**
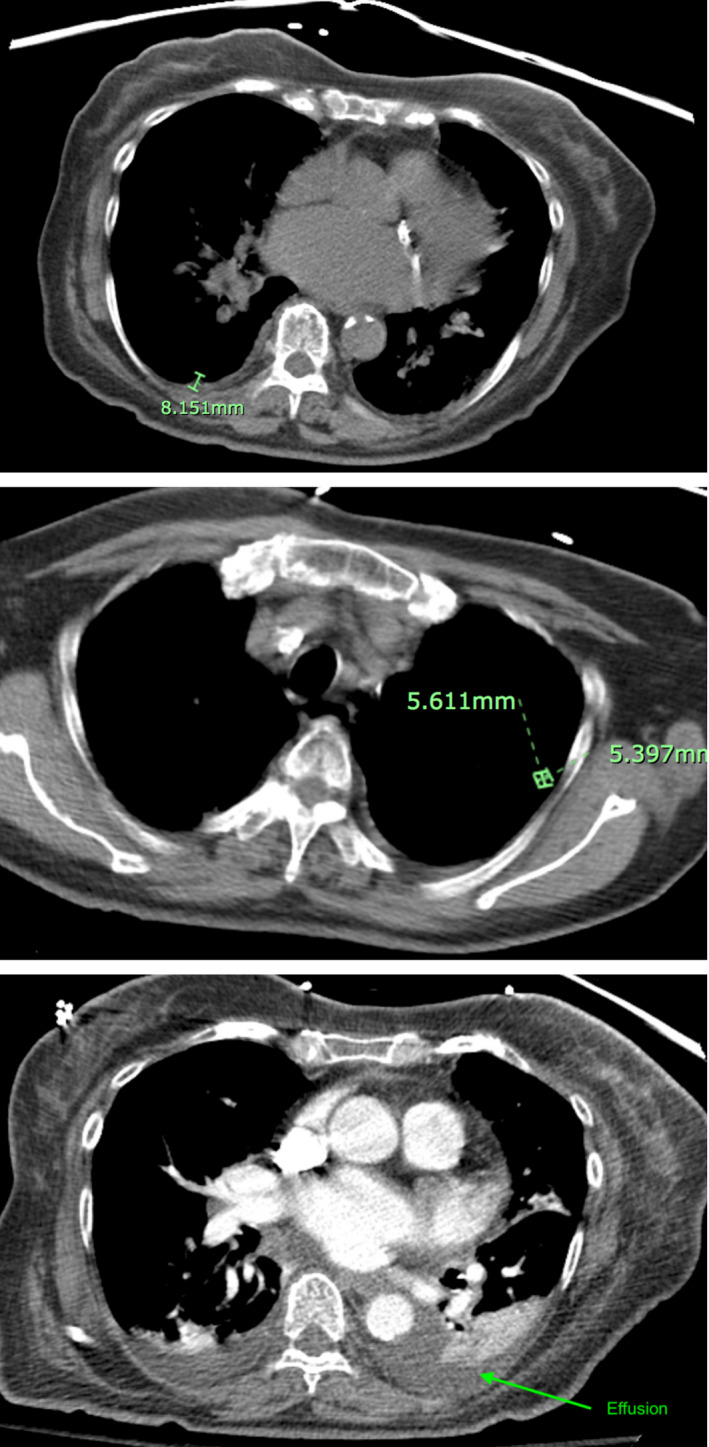
Chest CT scan without contrast showing 8.2 mm pleural nodule in right lung base (top), a 5.4 × 5.6 mm left upper lobe nodule (middle) and left lower lobe pleural effusion (bottom) that all together are suggestive of neoplasm

While in the emergency room, serial troponins were negative, D‐dimer, and BNP were within normal limits, and EKG was unremarkable. She had mild leukocytosis with elevated eosinophils and transaminitis, but an initial liver ultrasound was unremarkable (Table 1). She was started on 2 L oxygen via nasal cannula which improved her saturation to 94%. An initial chest x‐ray showed bibasilar infiltrates with small left‐sided effusion. A computer tomography (CT) scan of her chest showed left lower lobe consolidation, interstitial disease with fibrosis, vascular congestion, pleural nodule in right lung base (8.2 mm), and a left upper lobe nodule (5.4 × 5.6 mm) suggestive of neoplasm (Figure 1). She was admitted for acute respiratory failure with hypoxia and further investigation of pleuritic chest pain in the setting of a newly diagnosed lung mass.

**Table 1:** Initial ED labs on presentation

CBC with differential		General chemistry and coagulation	
WBC	12.9 × 10^3^/µL	Sodium	137 mEq/L
RBC	4.79 × 10^6^/µL	Potassium	3.2 mEq/L
Hemoglobin	14.6 g/dL	Chloride	99 mEq/L
Hematocrit	43.4%	CO_2_	24 mEq/L
MCV	90.6 fL	BUN	12 mg/dL
MCH	30.5 pg	Glucose	162 mg/dL
MCHC	33.6 g/dL	Calcium	9.9 mg/dL
RDW	11.9%	Creatinine, serum	0.68 mg/dL
Platelets	217 × 10^3^/µL	Total bilirubin	1.2 mg/dL
Abs neutrophil count	9843 cells/mm^3^	Bilirubin, Direct	0.31 mg/dL
Neutrophils relative	76.3%	Bilirubin indirect	0.89 mg/dL
Lymphocytes relative	6.7%	Albumin	4.3 g/dL
Monocytes relative	9.0%	Total protein	7.8 g/dL
Eosinophils relative	7.0%	Alkaline phosphatase	42 U/L
Basophils relative	0.5%	AST	139 U/L
IG relative	0.5%	ALT	57 U/L
Neutrophils absolute	9.9 × 10^3^/µL	Anion Gap	14 mEq/L
Lymphocytes absolute	0.9 × 10^3^/µL	eGFR non‐African‐American	>60 mL/min/1.732 m^2^
Monocytes absolute	1.2 × 10^3^/µL	Protime	12.5 s
Eosinophils absolute	0.91 × 10^3^/µL	INR	1.09
Basophils absolute	0.1 × 10^3^/µL	aPTT	26.4 s

Cardiac biomarkers		Urinalysis	
Total CK	46 U/L	Color	Amber
Troponin I	<0.03 ng/mL	Clarity	Hazy
BNP	71 pg/mL	Specific gravity	1.017
		Protein, ketone, glucose, bilirubin	All negative
Special chemistry		Blood	small
D‐dimer	419 ng/mL	Leukocyte, nitrite	Both negative
		Urobilinogen	Negative

## 3 INPATIENT INVESTIGATIONS

**FIGURE 2:**
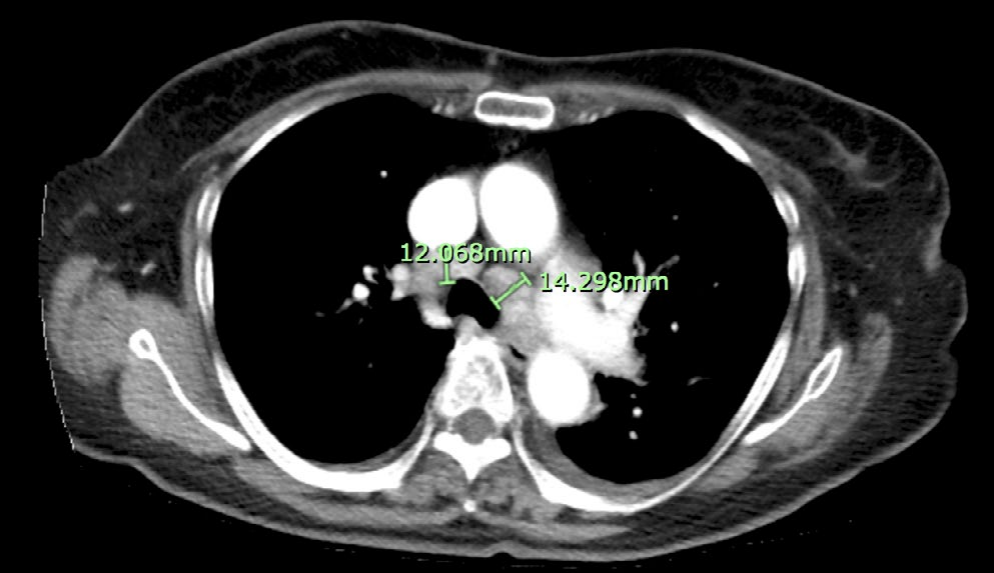
A follow‐up chest, abdomen, and pelvis CT scan with contrast showing multiple enlarged mediastinal lymph nodes without any evidence of a hilar or lung parenchymal mass

A follow‐up CT scan of her chest, abdomen, and pelvis with contrast showed enlarged mediastinal lymph nodes without any evidence of a hilar or lung parenchymal mass. (Figure 2) Pulmonology was consulted and a follow‐up endobronchial ultrasound (EBUS) guided biopsy was negative for lymphoma or other malignancy. Cardiology was also consulted and an echocardiogram showed a normal left ventricular ejection fraction and was otherwise unremarkable. Also, her pre‐existing UTI was being treated with ceftriaxone while inpatient.

On the third day following admission, the patient developed a fever and new‐onset atrial fibrillation for which she was started on rivaroxaban and an increased metoprolol dose. She also received doxycycline for suspected hospital‐acquired pneumonia given her fever and imaging findings. Shortly after, she developed a maculopapular rash across her abdomen and back while becoming hypoxic with increased work of breathing and 6 L oxygen demand. A CT angiogram of her chest ruled out a pulmonary embolism and showed bilateral pleural effusions. Antibiotics were broadened to piperacillin/tazobactam and vancomycin, and she was admitted to the intensive care unit (ICU).

## 4 NITROFURANTOIN‐INDUCED LUNG INJURY DIAGNOSIS AND TREATMENT

She had a 2‐day ICU stay where she was on BiLevel Positive Airway Pressure (BiPAP) support, supplemental oxygen, and IV furosemide diuresis. Infectious disease was consulted and given negative blood cultures, absence of clinical or radiological evidence of pneumonia, or a cardiac cause of fluid overload, a diagnosis of nitrofurantoin‐induced lung injury was suspected. This was supported by the patient's fever, worsening dyspnea, pleural effusion, new‐onset atrial fibrillation, erythema multiforme rash, and eosinophilia. All antibiotics, including ceftriaxone, piperacillin/tazobactam, vancomycin, and doxycycline were discontinued. The patient's symptoms improved, and she was gradually weaned off oxygen and downgraded. Despite improvement, she still had a 2 L O_2_ requirement with persistent eosinophilia and was started on high dose prednisone. Eosinophils dramatically improved and she was able to be weaned off O_2_ completely. She passed her home O_2_ evaluation and was discharged on 20 mg PO that was gradually tapered over 3 weeks, furosemide, and rivaroxaban in addition to her prior to admission medications. At her follow‐up 1 week after discharge, her eosinophil count and leukocyte count returned to within normal limits and her shortness of breath improved markedly. She continued showing improvement at subsequent monthly follow‐up visits and per her most recent follow‐up in July 2020 she remains symptom free.

## 5 DISCUSSION

Lung injury following nitrofurantoin use is a rare complication in the acute setting. Previous studies indicate the incidence of pulmonary injury due to nitrofurantoin use to be between 0.0001% and 0.001%, with 90% of these cases being acute.^4^ Patients with renal disease, older age (60‐70 years), and females are most at risk.^5^ Women represent 85%‐95% of the cases of lung injury due to nitrofurantoin and are accountable for almost all adverse reactions besides blood dyscrasia.^2^, ^6^ Women are more susceptible to UTI's, thus, are more likely to receive treatment with nitrofurantoin.^5^ Renal disease is significant because roughly 50%‐55% of the drug is removed unmetabolized via glomerular filtration and tubular secretion.^7^ Although a recent study found no difference in adverse reactions in women over 65 years with mild to moderate reduction in eGFR.^8^ Diagnosis is through exclusion. Given its rarity, patient workups predictably rule out more common or severe causes of lung injury such as pulmonary embolism, congestive heart failure, myocardial infarction, pneumonia, lung malignancy, COPD, ARDS, and others.^9^ This also occurred in our case.

For first‐time users, acute pulmonary reactions presented on average 8.7 days after nitrofurantoin use, 24 hours with repeat exposure.^10^ In our case, the patient presented with symptoms 2 days after beginning nitrofurantoin therapy with worsening of symptoms after 5 days. Patients typically present with the following symptoms in the acute setting: fever (82%), dyspnea (60%), irritating dry cough (43%), rash (20%), fatigue (12%), flu‐like symptoms (9%), cyanosis (4%), jaundice (3%), and weight loss (2%).^2^, ^11^ Pulmonary specific lung injury is associated with fever (80%), increased erythrocyte sedimentation rate (80%), eosinophilia (80%), and a dry cough (66%).^12^ Our patient experienced chest pain, fever, rash, and eosinophilia, typical of other known reports. It is worth noting it is unknown whether the patient had any previous history of nitrofurantoin use. It is also possible the patient suffered an allergic reaction to ceftriaxone or doxycycline but highly unlikely. She was experiencing symptoms before these two drugs were administered, had previous use of ceftriaxone without issues, and her rash was not consistent with that of doxycycline use. Furthermore, our patient did not fully satisfy any known diagnostic criteria for DRESS syndrome such as the RegiSCAR, Bocquet's criteria, or Japanese‐induced hypersensitivity syndrome (DIHS) criteria.^13^ Also, given the rarity of DRESS syndrome and limited evidence of association with nitrofurantoin, nitrofurantoin‐induced lung injury was the most likely diagnosis for this case.

Physical exam and imaging for nitrofurantoin‐induced lung injury are nonspecific. Patients may show respiratory distress, rales, crackles, or nothing on physical exam.^14^ Chest x‐ray may be normal or reveal bilateral lower lobe interstitial infiltrates with or without pleural effusions.^14^ Chest CT typically will show bilateral ground glass opacities.^10^ Other comorbidities may convolute the findings of imaging. For example, on chest x‐ray both pulmonary edema due to heart failure and nitrofurantoin‐induced lung injury look very similar. Chest x‐ray and CT scan findings in our patient were consistent with expected imaging. Pathophysiology of nitrofurantoin‐induced lung injury is multifaceted. Studies have indicated the following mechanisms: interstitial inflammation, reactive type II pneumocytes, vasculitis, eosinophilia, focal hemorrhage, small organizing microthrombi, and alveolar exudates associated with macrophages.^5^, ^10^, ^15^, ^16^, ^17^ These mechanisms are possibly initiated via oxidation reactions in the lung. Sasame et al demonstrated via an in vivo study that there was a reduction in injury to lung tissue when nitrofurantoin was administered with Vitamin C compared to nitrofurantoin alone.^17^ This mechanism is not entirely understood, but it might be due to Vitamin C's antioxidant properties and its ability to negate the products of nitrofurantoin‐induced superoxide production, hydrogen peroxide production, and nicotinamide adenine dinucleotide phosphate (NADPH) oxidation.^10^, ^15^, ^17^

Treatment for nitrofurantoin‐induced lung injury in the acute setting is the discontinuation of the drug. Patients experience improvement of symptoms in a relatively short time period, days to weeks.^10^, ^12^ A common treatment for nitrofurantoin‐induced lung injury is the use of glucocorticoids. Glucocorticoids have not been proven to be effective in changing prognosis or decreasing recovery time.^5^ One caveat to this may be that most studies about glucocorticoid use in the context of lung injury from nitrofurantoin use are in the chronic setting. More studies need to be conducted in the acute setting. In this case, the patient's persistent need for O_2_ and eosinophilia following nitrofurantoin use were indications to start the patient on high dose prednisone. After the addition of the prednisone the patient O_2_ requirements were weaned off and eosinophil levels decreased to normal levels. It is unclear whether this was due to more time following nitrofurantoin discontinuation or as a result of the glucocorticoid.

## 6 CONCLUSION

Nitrofurantoin has been proven to be an effective first‐line antibiotic for the treatment of lower UTI in the general population. In this particular case, our patient most likely suffered from a rare but serious adverse effect of nitrofurantoin therapy leading to a presentation of acute lung injury. With the initiation of nitrofurantoin 2 days prior to presentation as well as the exclusion of other possible etiologies such as pulmonary embolism, malignancy, and myocardial infarction, this points to nitrofurantoin as the most likely causative agent. Although the mechanism behind the cause of acute lung injury is still unclear, the management of this rare presentation includes discontinuation of nitrofurantoin with possible initiation of oral glucocorticoids if there is suspicion of respiratory distress.

## CONFLICT OF INTEREST

The authors declare no conflict of interest.

## AUTHOR CONTRIBUTIONS

RK: initiated this case report; performed literature review and wrote the final manuscript draft. KS: performed literature review and contributed substantially to this manuscript's contents. IS: reviewed the manuscript for intellectual content and contributed key content. KS: advised authors and reviewed this manuscript for intellectual content.

## ETHICAL APPROVAL

This case report does not contain any clinical studies with human participants performed by any of the authors.

## CONSENT

Patient provided written consent for the publication of this case report.
